# A Smart Sensing System of Water Quality and Intake Monitoring for Livestock and Wild Animals

**DOI:** 10.3390/s21082885

**Published:** 2021-04-20

**Authors:** Wei Tang, Amin Biglari, Ryan Ebarb, Tee Pickett, Samuel Smallidge, Marcy Ward

**Affiliations:** 1 Klipsch School of Electrical and Computer Engineering, New Mexico State University, 1125 Frenger Mall, Las Cruces, NM 88003, USA; aminb@nmsu.edu (A.B.); rlebarb@nmsu.edu (R.E.); 2 College of Agricultural, Consumer, and Environmental Sciences, New Mexico State University, 940 College Dr, Las Cruces, NM 88003, USA; tpickett@nmsu.edu (T.P.); ssmallid@nmsu.edu (S.S.); maward@ad.nmsu.edu (M.W.)

**Keywords:** RFID, water intake, animal agriculture, motion detector, water quality, watering behavior, ranch, livestock, wildlife, data acquisition

## Abstract

This paper presents a water intake monitoring system for animal agriculture that tracks individual animal watering behavior, water quality, and water consumption. The system is deployed in an outdoor environment to reach remote areas. The proposed system integrates motion detectors, cameras, water level sensors, flow meters, Radio-Frequency Identification (RFID) systems, and water temperature sensors. The data collection and control are performed using Arduino microcontrollers with custom-designed circuit boards. The data associated with each drinking event are water consumption, water temperature, drinking duration, animal identification, and pictures. The data and pictures are automatically stored on Secure Digital (SD) cards. The prototypes are deployed in a remote grazing site located in Tucumcari, New Mexico, USA. The system can be used to perform water consumption and watering behavior studies of both domestic animals and wild animals. The current system automatically records the drinking behavior of 29 cows in a two-week duration in the remote ranch.

## 1. Introduction

Water is an essential resource in animal agriculture and the number one nutrient required by ruminants [1]. Animal agriculture utilizes 30% of the total water in food production [2]. Given water’s potential impact and a recognized need for improved technologies in rangeland agriculture [3,4], farmers and ranchers continually seek ways to optimize their water management. This need has incentivized the development and utilization of innovations in agriculture. In drought-prone areas such as the southwestern United States, spatial and temporal variation in water availability results in reductions in forage quality and quantity, affects animal productivity, and influences natural resources management decisions. For example, in 2014, livestock producers proactively reduced their herds in response to drought, resulting in New Mexico beef cow inventories dropping by 53,000 cows [5]. In order to remain profitable under variable environmental conditions, livestock producers whose herds are efficient in feed and water use or who adopt water intake efficiency monitoring technologies to inform management decisions will improve fiscal resilience. Therefore, it is important to understand animal water consumption behavior based on both water quality and water access.

Numerous predictive equations have been proposed related to water consumption in beef cattle. For example, in 1956, Winchester and Morris authored a paper evaluating the effects of water intake from dry matter intake, air temperature, and bovine genus [6]. Since then, several equations have used additional variables such as salt supplementation, body weight, milk production, humidity, and wind speed [1,7,8,9,10]. Dry matter intake, air temperature, and body weight are the most common variables used to create these models. However, these methods along with many data sets were developed from cattle in confined situations, which do not represent the southwestern desert ecoregion [11]. Despite multiple water intake parameters being utilized in prediction models, water intake estimates vary greatly and have limited applications for producers in the US southwest. For instance, when a 489 kg lactating cow and standardized variable values are used, predicted water intakes range from 37 to 83 L per day per animal. Other variables not considered in these models are distance to water, climate, and elevation. Breed of cattle likely could have an impact on water intake, watering behavior, and efficiency of use. However, there are currently no data available on these variables related to cattle grazing large pastures greater than 80 ha, while typical pasture sizes in arid regions of the desert southwestern US average over 4000 ha.

So far, little has been accomplished in quantifying individual animal variability and efficiency of water intake in rangeland environments. In recent years, a limited amount of studies have attempted to address the genetic influence on water consumption in beef cattle [9,10,12]. Nevertheless, the technologies utilized in these studies were limited to confinement situations. In addition to domestic cattle, water consumption by feral animals and wildlife contribute another dynamic to the agroecosystems. Currently, direct quantification of water intake by wild large ungulates [13] or other wildlife species is limited. For example, extant estimates of water consumption by wild ungulates (e.g., Rocky Mountain elk, *Cervus elaphus*) rely on formulas developed for domestic animals [11], are based on confined animals [14], or use indices [15]. Improved knowledge of water requirements for wild animals under variable natural environmental conditions will contribute to wildlife conservation planning significantly. Quantified information on water intake by wildlife will contribute to a debate on the value of anthropogenic water developments for wildlife and the effects of supplemental water in the environment [16,17,18,19,20,21]. Additionally, portable water intake technology would provide new insights to wildlife habitat relationships, population dynamics, and response to variable environmental conditions. A primary reason for limited information on wildlife water intake is a lack of technology available to quantify water intake in remote areas that will not disrupt natural watering behaviors.

To address the above-mentioned challenges, there is a significant need for an intelligent drinking system that refills the drinking tanks as well as records the quantity and quality of water consumed by each identifiable individual animal. Currently, there are very few solutions addressing water management for monitoring watering behaviors for animal agriculture. Commercial solutions, such as Insetec from Hokomfarm, are usually expensive and mainly utilized by larger framing corporations or research institutions, whereas smaller-sized ranching operations cannot afford to implement these systems for their drinking stations. Another issue is that commercially available solutions are usually confinement setups, which are not easy for remote grazing sites and not able to be used for monitoring wild animals. Based on the design constraints, biological sensing and data acquisition systems [22,23,24,25] would take an important role in developing an integrated water monitoring system by combining low-cost sensors, embedded systems, and data communication or storage devices [26,27,28,29,30,31,32]. Such devices are expected to be deployed in the remote ranching sites to track the water intake and watering behavior of both livestock and wildlife.

**Figure 1:**
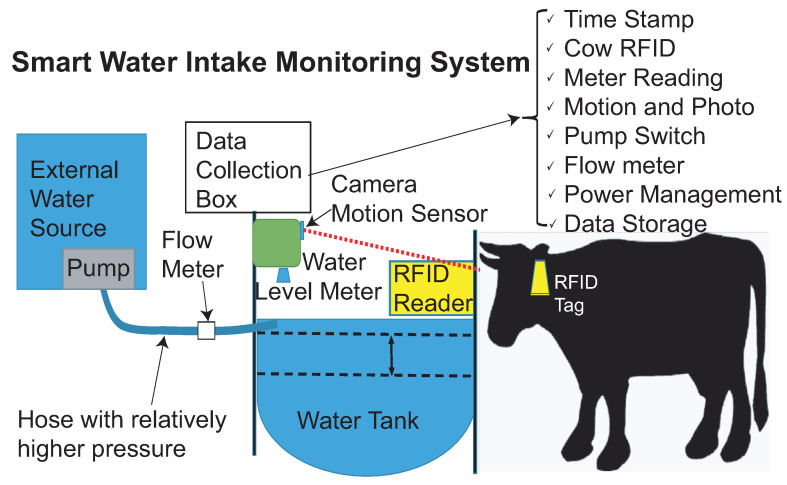
The proposed smart water intake monitoring system with multiple sensors and data collections.

Recently, Radio-Frequency Identification (RFID) technologies [33] have gained popularity in the application of the internet of things [34,35] and especially in sensors and sensing systems [36,37], such as 3D chipless RFID tags [38], fully-textile chipless tags [39], and frequency-signature based wearable tags [40]. The wireless sensor network has also been playing an important role in industrial and agricultural applications [41]. In this work, we propose a portable smart water intake monitoring system for measuring and analyzing the quantity and quality of the water of livestock and wildlife in remote locations. The domestic livestock includes cows and sheep, and the wildlife animals include wild horses and deer. The system is based on multisensor integration in a cost-effective and affordable manner using the complementary method, as shown in Figure 1. The sensors used in the system include RFID readers, motion detectors, water level meters, water flow meters, and temperature sensors. In this system, the RFID reader is applied to identify tags attached to cattle that use the system. The motion detector activates the RFID reader, the camera, as well as the water level meter and water flow meter once an animal, domestic or wild, moves in front of the drinking station. The camera records the front image of the animal. The system records the water amount that the specific animal consumes each time as well as the drinking duration. As wild animals do not have an RFID tag, water consumption by wild animals is recorded using the motion detector and the camera. The water level meter measures water consumption in the drinking station tank. When the level of water in the tank falls below a specific amount, the system opens a valve and refills the tank with water from an external water source, which is recorded by the water flow meter. All the data recorded by the system are stored on an SD card. The system is automated and functions without user input once set up in the field. The sensor network method [41] plays an important role in the proposed system. Compared with other similar systems mentioned in the previous paragraph, the proposed system utilizes sensor networks that combine vision sensors, motion detectors, RFID systems, flow meters, temperature sensors, and turbidity sensors. The complementary data from individual sensors provide a reliable and accurate recording of water quality and water intake of individual animals.

This paper focuses on a water intake monitoring and measurement system for animal agriculture. The main innovation and contributions of this paper include the (1) development and implementation of the proposed system on both the experimental and real field application, and (2) validation of the system functionality and measurement of the performance of the system for both the domestic cattle and wild animals. The paper is organized as follows. Section 2 provides the system design details including both the hardware architecture and software control flow. Section 3 presents the experimental setup and application considerations. Section 4 illustrates the experimental results and data analysis. Section 5 discusses the advantages and shortcomings of the current system. Section 6 concludes this paper.

## 2. System Design and Implementation

The main job of the system is to perform a serial of functions to correctly monitor the water quality and water consumption for both domestic livestock and wild animals. For example, the system should detect the event that the animal is at the trough, which is about 60 cm away from the motion detector. Furthermore, the system should be able to identify the specific livestock using the RFID ear tag. The system needs to take pictures when any animal visits the drinking station. In addition, the system should be able to measure the amount of water that the animal drinks each time, as well as the temperature and turbidity of water. The data, including water consumption, water quality, timestamp, animal identification (ID), and pictures, should be stored in an SD card. Moreover, the system must trigger a warning signal to indicate if the water level is too low. Above all, the system must be able to withstand harsh environments.

### 2.1. Power Distribution and Data Communication

**Figure 2:**
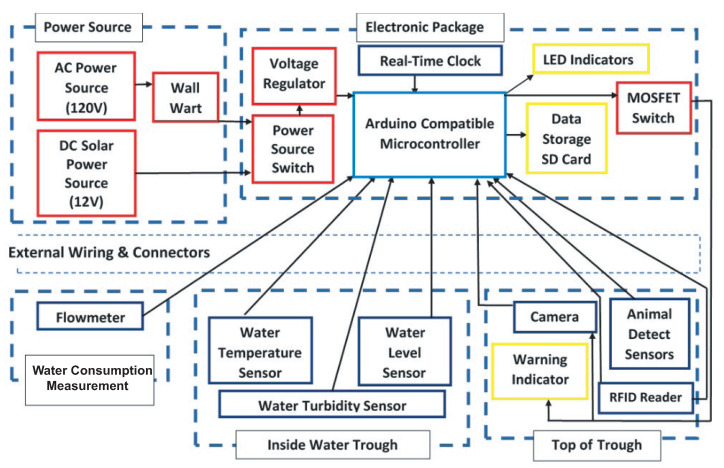
Hardware block diagram of the smart water intake monitoring system.

The major design considerations of the water intake monitoring system are the power distribution and data communication from multiple sensors. The system has two power interfaces: one is 12 V Direct Current (DC) from the solar panel and the other is 120 V Alternating Current (AC) from the AC power source. A power source automatically selects the AC source if the AC source is available; otherwise, the system uses DC solar power. The AC power goes through a power adapter which turns the voltage into 12 V DC. The 12 V DC source powers the Microcontroller Unit (MCU) board. A voltage regulator that can handle up to 1A converts the 12 V DC to 5 V DC that is usable by the electronic components. The electronic components that connect the MCU board include a Real-Time Clock (RTC), an SD card for data storage, two Light-Emitting Diode (LED) light indicators, and three Metal Oxide Semiconductor Field Effect Transistor (MOSFET) switches. These components are hosted in an electronic box with the MCU. The MOSFET switches are controlled by the MCU to power the warning lights, the Top of Trough sensors, and the Inside Water Trough sensors. The Top of Trough sensors are the RFID Reader, the Camera, and the Motion Detector sensor to detect the animal visits. The Inside Water Trough sensors are deployed inside the water trough, including the temperature sensor, the water level sensor, and the water turbidity sensor. The RTC controls the timing information of the overall system. The RTC communicates with the MCU via the Inter-Integrated Circuit (I2C) bus and the SD card via the Serial Peripheral Interface (SPI) bus. The external wiring and connectors connect the electronics packaging to the sensors. The system also has a water measurement unit, which is a water flowmeter that is in line with the water line that feeds water to the trough. The overall water sensing system is shown in Figure 2.

The overall sensing system can be divided into one central control subsystem and two sensing subsystems. The central control system has an Arduino Mega 2560 MCU, the RTC, and the Micro SD Card adapter. The Arduino Mega 2560 is the central processing unit of the system. It is connected to all the components of the system through a custom-designed Printed Circuit Board (PCB) and ethernet cable. All the software and signal processing of the system is also executed on the Arduino Mega. One of the sensing subsystems is for animal sensing and the other is for water sensing. The individual component implemented in the animal sensing subsystem includes an RFID tag reader and a weatherproof Adafruit TTL Serial JPEG Camera with the motion detector. The RFID reader identifies individual cows when the RFID tag approaches the drinking station. The RFID tags are attached to the ears of the cows. During each drinking event, the ID numbers of the RFID tag are recorded along with the amount of time spent on the drinking station as well as the volume of water consumed. The weatherproof TTL camera visually records the animals drinking by taking several pictures of them. The camera uses a built-in motion detector to activate and start recording. This can sometimes cause issues such as the movements of the objects in front of the camera. Sometimes, even strong winds can cause the camera to activate and take pictures, even though none of the livestock is using the drinking station. This problem is solved using the RFID system by only allowing the images taken by the camera during the RFID event to be stored in the SD card. The water sensing subsystem has a temperature sensor, a sonar sensor, a water flow meter, and a water turbidity sensor. The sonar sensor measures the depth of the water within the water tank. Should the water level drop beneath a specific amount, the sensor would contact the CPU, which would open the flow meter and refill the water tank to raise the water volume to ensure animal welfare.

### 2.2. Hardware and Software Implementation

The hardware of the sensing system contains the SD Card Adapter, the motion detector with the camera, the thermal sensor, and the RFID Reader. The SD Card Adapter is the system’s data storage, all the images, RFID tags, thermal readings, and timestamps taken by the other components of the system are stored on a single SD card slotted on to this adapter. In order to access the recorded data, users must manually remove the SD card and insert it into a device. The thermal sensor measures the temperature of the water in the drinking station’s tank. The RFID Tag Reader reads the tag on the ears of the animals on the field and identifies them. It allows the system to match the recorded data of the volume of water consumed and the amount of time spent on the drinking station by each animal. The RFID Reader used in the smart sensing system is a Nordson NK-RF12 125 kHz long-range reader. It is a 125 kHz, RS-232 standard reader with a 9–16 V operating voltage and 70–100 cm reading range. Each unit weighs 1.5 kg and has a size of 230×230×35 mm. To operate, the reader is connected to a power source and the overall system through its VCC and GND, and RxD and TxD cables, respectively. The RFID tag [42] is another important component of the system. In our system, the RFID tags were passive RFID tags with a rectangle shape and a size of 8.5 cm by 5.4 cm that has a read distance of approximately 30 cm.

The operation of the RFID reader is simple. Every time a compatible RFID tag is passed by the reader, the reader reads its ID and transmits the data to the system. The system then saves the ID alongside a timestamp and date of the detection in an excel file format within the SD card data storage. As the Reader operates on a typical RS-232, 9–16 V voltage range, its output cannot be connected directly to the Arduino Mega, as any input above 5 V can damage the Arduino board. Therefore, the reader’s output is first passed through an RS232 to TTL converter which in turn is connected to the Arduino board. The functionality of the RFID Reader is first tested by attaching it alone to the Arduino board and running a simple RFID Reader code to ensure that it can read tag IDs and send them to the board. The simple testing code utilizes a custom library and sets the default pins for the Arduino Mega’s respective RxD and TxD pins for both receiving and transmitting data from and to the RFID reader. The scanned ID number is converted into a hexadecimal number and saved to the CSV file in the SD card alongside the time and date.

**Figure 3:**
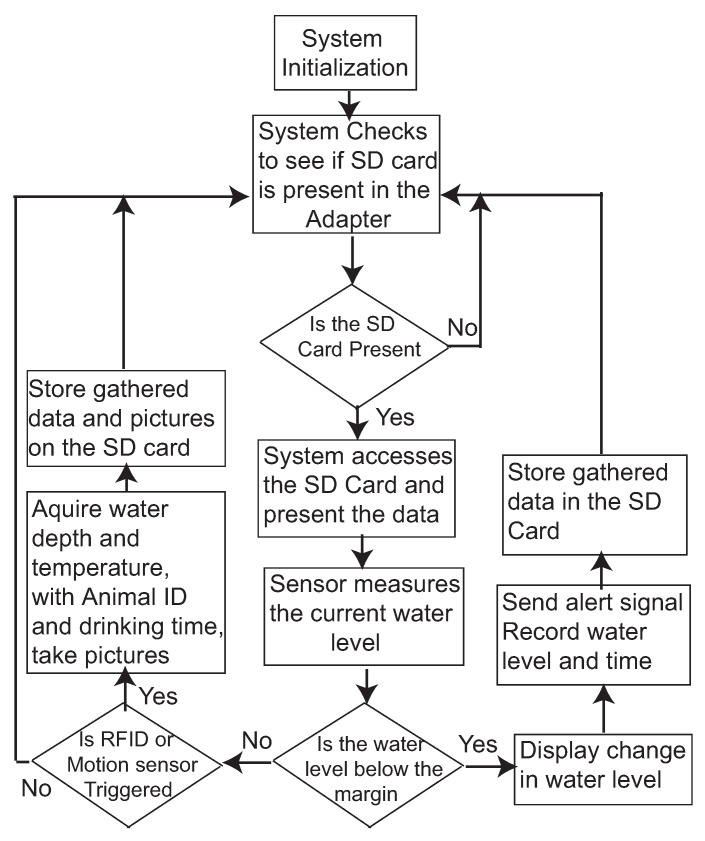
The flow chart of the control program for sensing, processing, and data storage.

The system software control flow chart is illustrated in Figure 3. After system initiation, the system checks if the connection of the SD card is in a good shape, then it measures the water level to make sure that there is enough water for the animal. If there is not enough water, the system sends an alert and displays the water level on an LCD screen. The water level information is then stored in the SD card. If the system passes the initial checkups, the system searches events from the RFID reader and motion detector, and if there is a drinking event, the system records the data of the event in terms of water depth, temperature, and animal ID. The control program is running on the Arduino Mega board. The current control flow chart uses a polling method to check the RFID and Motion detector every time. The algorithm efficiency could be improved by adapting the interrupt method to let the RFID or Motion Detector to trigger the system data collection.

### 2.3. Water Consumption Estimation

To best create a situation that does not inhibit watering behavior in livestock or wildlife, the system has up to multiple individual water troughs to provide adequate access and volume to collect data from multiple animals simultaneously. Calculating the change in volume during a drinking event is the best option to determine water consumption. In the experiment site, each trough in the drinking station has a 416 L capacity. The dimensions of these troughs are 124.5 cm in length, 84 cm in width, and 53 cm in depth. Equation (1) describes the formula utilized to calculate consumption ΔV:

(1) $$\Delta V=\frac{\pi \cdot a\cdot b\cdot \left(h_{1}-h_{2}\right)}{1000}$$

where *a* is the short axis, which is half of the width, and *b* is the long axis, which is half of the length. Change in depth is determined, where $h_{1}$ is the height reading at the end of a drinking event and $h_{2}$ represents the height reading at the beginning of the drinking event. Equation (1) is applied to the system to measure the water consumption based on the readings of the water level sensors. The distance sensor has a resolution of 0.1 cm. As the animal drinks, the sensor records the increased distance from the sensor to the surface of the water. Based on the above parameters, the conversion based on this formula is that every 0.1 cm change in depth equates to 0.82 L consumed.

## 3. Experiment Setup

Inputs of the remote water sensing system were water level and flow measurements, water temperature, camera and RFID for animal identification, and proximity/motion detector. Specific outputs of the system are all data from the sensors store on the SD card, the water valve control relays (if troughs not gravity-fed), and a flashing light to serve as a malfunction indicator if something on the system were to fail. The systems are primarily located in remote locations far from any power sources so they need to be able to operate on DC solar power. In our experiments, the systems were deployed in two locations. The first system was implemented in a testing site in Corona, New Mexico, USA for functional validation, and other systems were deployed in Tucumcari, New Mexico, and USA for real application and data collection. Both systems are working in outdoor conditions.

**Figure 4:**
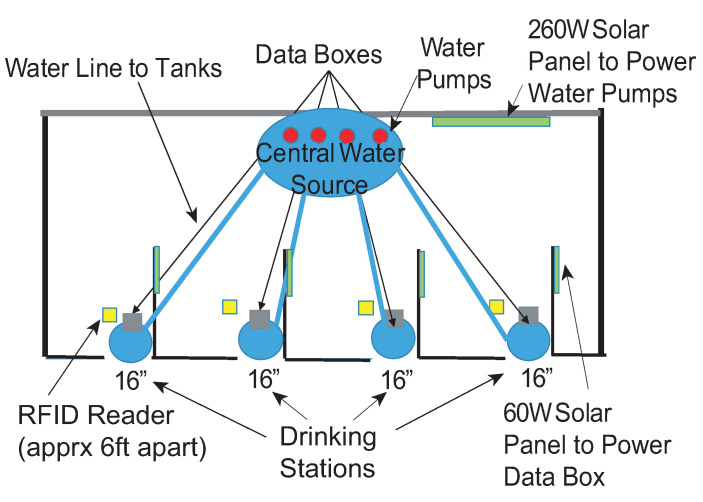
Drinking station deployed in the testing site.

**Figure 5:**
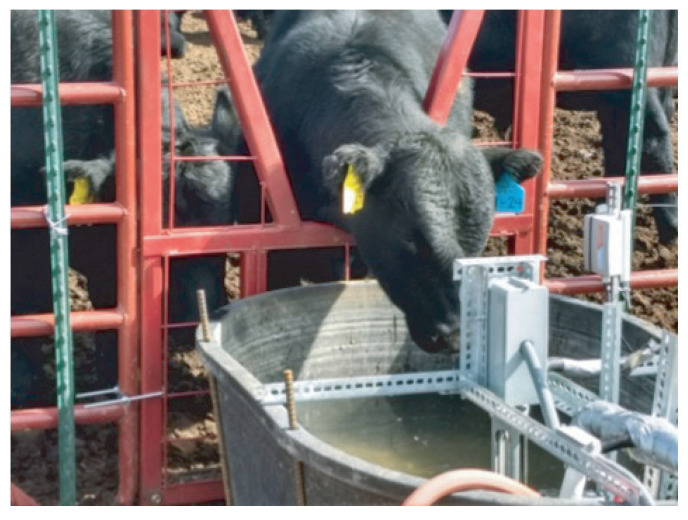
Drinking station with a cow drinking from the trough.

**Figure 6:**
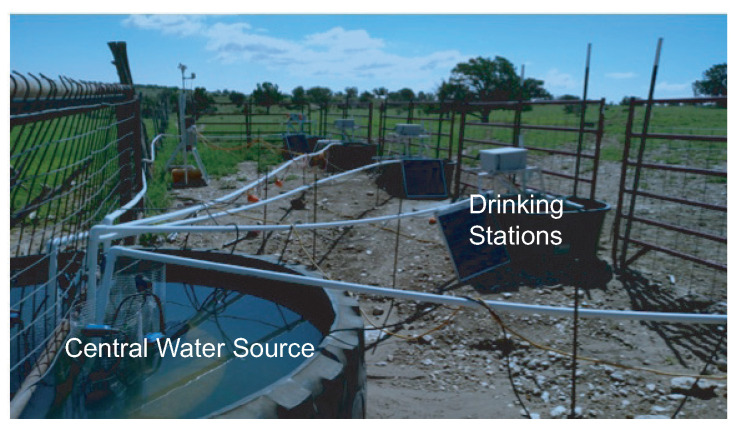
System setup at a remote testing site with drinking stations and a central water source.

The main goals of the system are to (1) identify each animal by RFID ear tag and/or photograph, (2) measure the water consumption of each of those animals, and (3) measure the quality of the water. The system schematic in Corona is illustrated in Figure 4. The central water source supplies four drinking stations. The central water source uses a dedicated water pump to control the water amount in each drinking station. The distance between the neighboring drinking stations is about 6 feet. Solar power is applied to support the electronics at the central station and each drinking station. Each drinking station has a trough and an opening for the cow as shown in Figure 5. Only one cow could drink in the trough at one time. The overall system implemented in the Corona testing site is shown in Figure 6.

**Figure 7:**
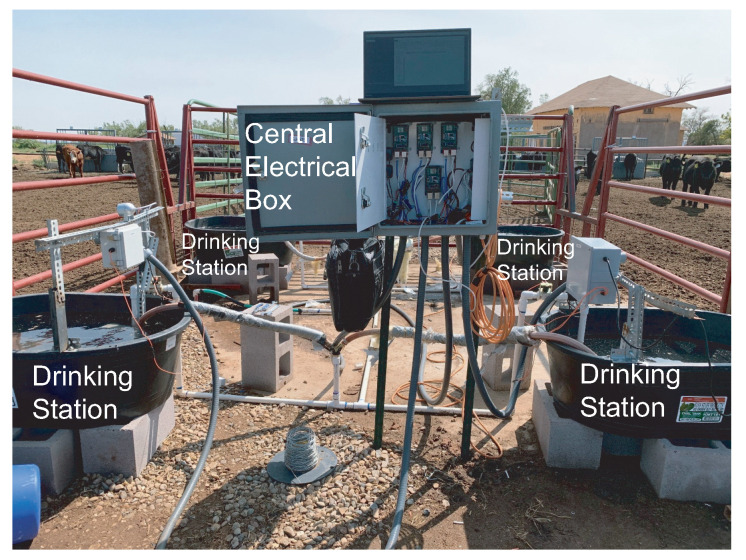
Setup at system test site with a central electrical box and four drinking stations.

**Figure 8:**
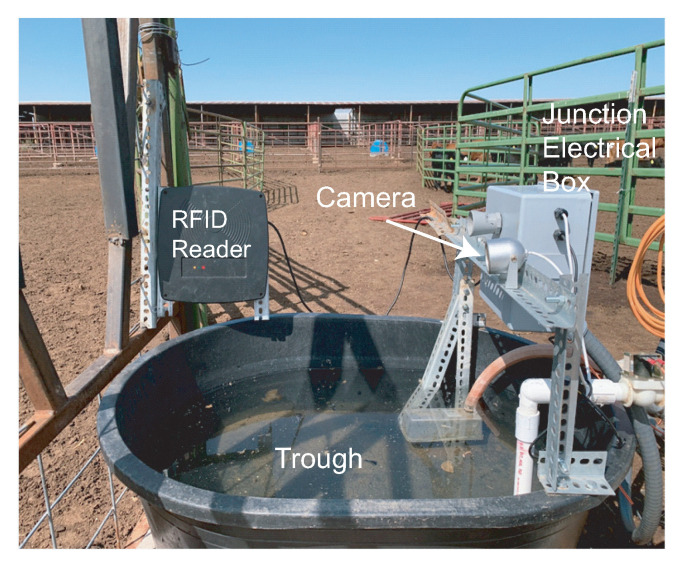
The side view of the drinking station showing the Radio-Frequency Identification (RFID) reader and the motion detector triggered camera.

**Figure 9:**
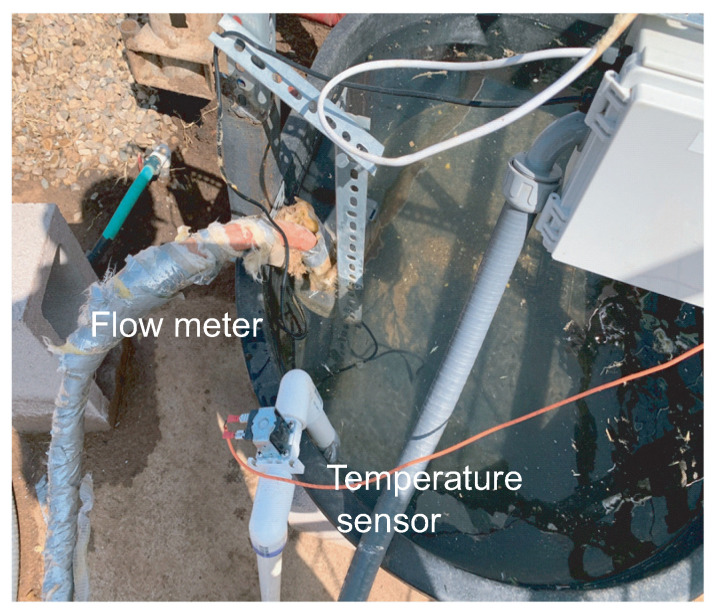
The flow meter and temperature sensor deployed in the trough.

**Figure 10:**
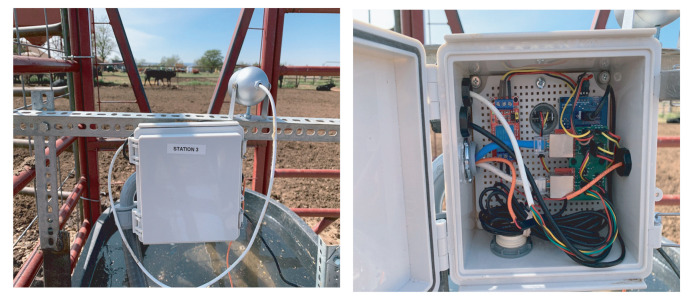
The junction electrical box implemented in each drinking station.

**Figure 11:**
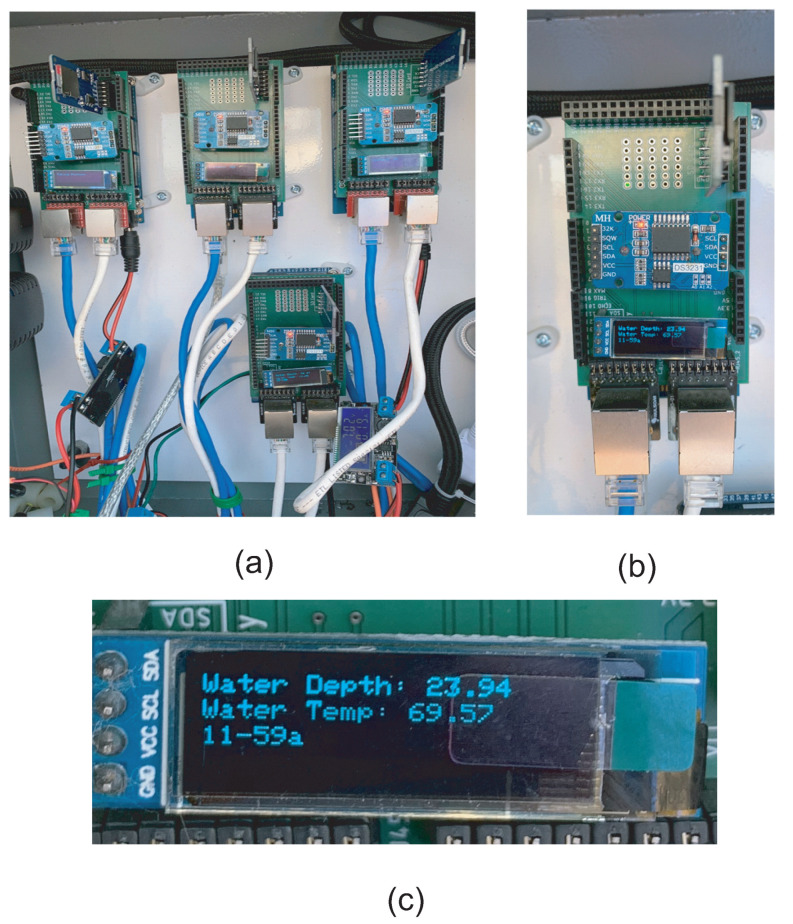
The central electrical box implemented for four drinking stations. (**a**) Four Arduino boards for each drinking station. (**b**) An Arduino boards with breakout boards. (**c**) Water information display on an LCD screen.

The data collection system deployment in the Tucumcari site is illustrated in Figure 7, Figure 8, Figure 9, Figure 10 and Figure 11. The Tucumcari site is in a more controlled setting, allowing for easier field troubleshooting during the development phase of the system. Figure 7 illustrates one of the systems deployed in the Tucumcari site. The system has one central sensor box which contains four Arduino Boards and four drinking stations that are connected to them. The four Arduino boards act as the central processing unit of each drinking station. It collects and stores data from its different components. Each Arduino board is connected to a junction box containing a converter board. The junction box is connected to the flow meter that tracks the total amount of water. One thermal sensor is deployed at each drinking station to record the water temperature data during the drinking events. Furthermore, each drinking station has an RFID Reader to read the ID of the individual animal’s RFID Tag. A camera is also implemented on each drinking station to take pictures once the motion detector is triggered when an animal is visiting the drinking station.

Figure 8 shows a sideway look at one of the system’s drinking stations. The black tub on the bottom is where the animals drink their water from. The RFID reader is visible on the left side of the water tub. The RFID reader reads the tags on the domestic animals’ ears as they extend their heads to drink. The camera with the motion detection sensor is screwed on top of metal bars on the opposite side of the animal’s head and starts recording the drinking event when it detects any motion. The Camera is screwed on top of metal bars on the opposite side of the animal’s head and starts recording the drinking event when it detects any motion. The flow meter and the thermal sensor are both located below the camera and measure the amount of water poured into the tub and the water temperature, respectively. A water level sensor is deployed on the bottom of the metal bar. It senses the distance between the sensor and the water level of the tub in order to obtain the water intake information.

Figure 9 shows a closeup of the drinking stations flow meter, which is the silver-taped pipe on the top of the picture, and the thermal sensor, which is the white pipe on the bottom of the picture. The flowmeter tracks animal water intake by refilling the station’s water if the water level is below a predetermined threshold. The thermal sensor keeps constant track of the water temperature and records the value for each drinking event. Figure 10 shows the external deployment and internal structure of the junction box of one of the drinking stations. A converter board is visible on the right side of the box. It is plugged into two ethernet cables and several wires. The board is responsible for the transference of data from the sensors and camera to the Arduino Mega boards in the central circuit box; it is connected to the components via the connected wires on the right side of the board and to the Arduino Mega via the ethernet cables on the left side of it.

The internal structure of the central circuit box is illustrated in Figure 11. Figure 11a shows the central electrical enclosure containing all the system’s Arduino Mega 2560 boards. Each board acts as the CPU of an individual drinking station. The main function includes running the operational code for each of the different components of the station, receiving the output signals from the sensors, and storing the outputs on an SD card. The SD card can store the data for more than two weeks, so the user does not need to fetch data very often. Figure 11b shows a closeup of one of the systems Arduino Mega 2560 boards. The board hosts breakout boards for an SD card, a DS3231 RTC for clock signal generation, and an LED display. The Arduino Mega operates the program for each of the drinking stations components including the camera, the thermal sensor, the flow meter, and the RFID reader. All the collected data are stored in the SD card for each drinking event. The RTC is used for tracking time and providing the time of each drinking session’s beginning and end. Each board also has an LCD display screen, which shows all the current properties of the water as well as the time for the users to observe directly without having to open the SD card, as illustrated in Figure 11c.

There are several additional and important requirements for the experiment setup. The first is that the system should be designed to let the livestock always have access to water even if the sensing system failed. The second is that the system should be able to withstand the harsh outdoor environment of the remote site for a long time during the experiments and data collection period. Minimum maintenance efforts are expected as the livestock experts who visit the site frequently may not be trained for operating the electrical sensing system.

## 4. Experiment Results

The system was deployed in Tucumcari, New Mexico, in the summer of 2020. Two individual systems were placed on both the east side and the west side of the remote station. The system records the drinking events in terms of date, time, water temperature, and initial and final depth of water level. Sensor data and animal behavior data are collected every two weeks. The system senses the animal visit each time at the drinking station and records the water intake. The water intake is then calculated by the difference of the distance sensor readings using the water consumption estimation equation. Animal identifications were captured using both the RFID system and the motion camera system. Water temperature is also recorded. The water turbidity sensor was not enabled and would be added in the later experiments. The main components of the system—animal identification, water intake measurements, and water temperature sensor—are working as expected. The current consumption of the system is 150 mA. Average watering behavior trends and individual animal drinking behavior data were collected and analyzed. The detailed experiment results are presented below.

**Figure 12:**
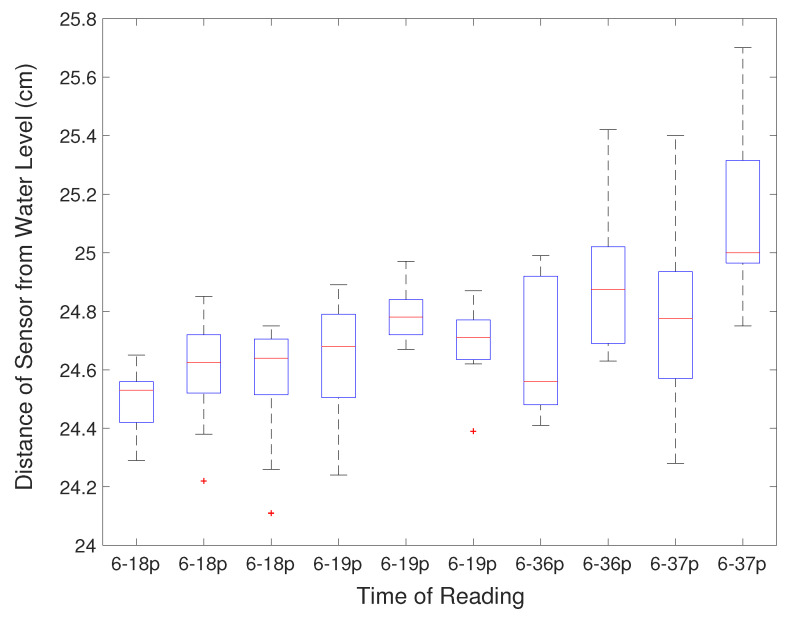
Statistical box plot of the water level sensor readings.

The water intake is measured by the water level using the distance sensor. Due to the fluctuation of the water level, each reading of the sensor may not accurately reflect the real water level. Therefore, the system samples the water level multiple times during each drinking event. Statistical methods were used to estimate the actual water level. Figure 12 shows the box plot with errors of the distance sensor readings over time. Only the average water level from multiple sensor readings was recorded in the SD card as the final data, while other readings were discarded in order to save the total data size. The water level estimation is done automatically in the Arduino microcontroller. The plot shows that the average distance of the water level increases, which means the water level is moving away from the fixed sensor due to water consumption. The total sum of the data from the water intake measurement system agrees with the data collected from the flow meter which measures the total water consumption.

**Figure 13:**
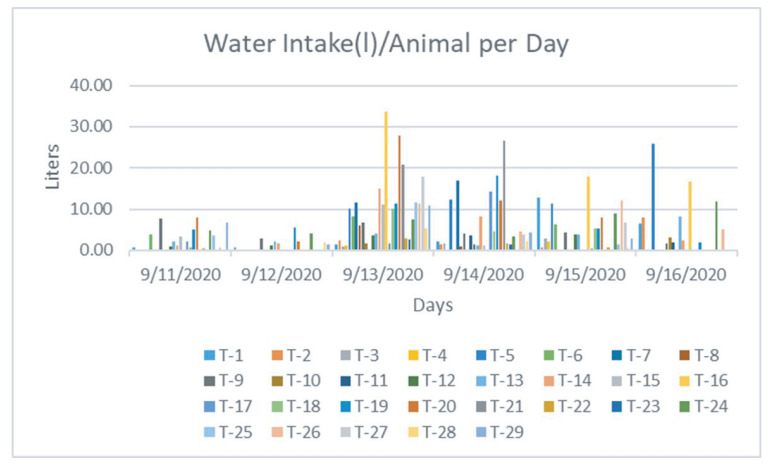
Recorded water intake data by each individual cow.

**Figure 14:**
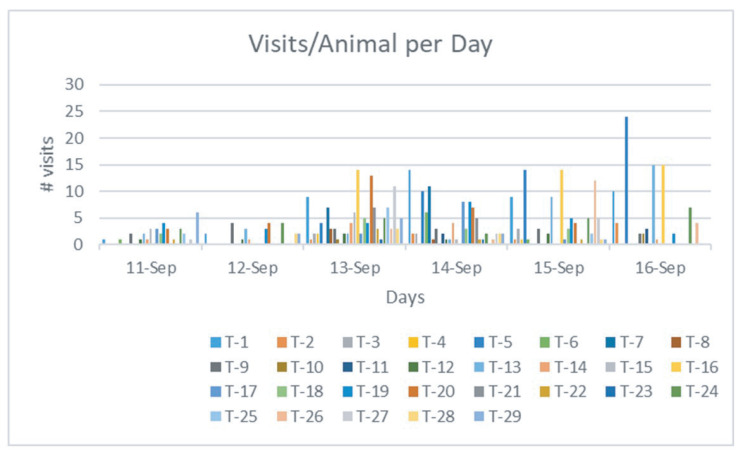
Recorded visits of the drinking stations by each individual cow.

**Figure 15:**
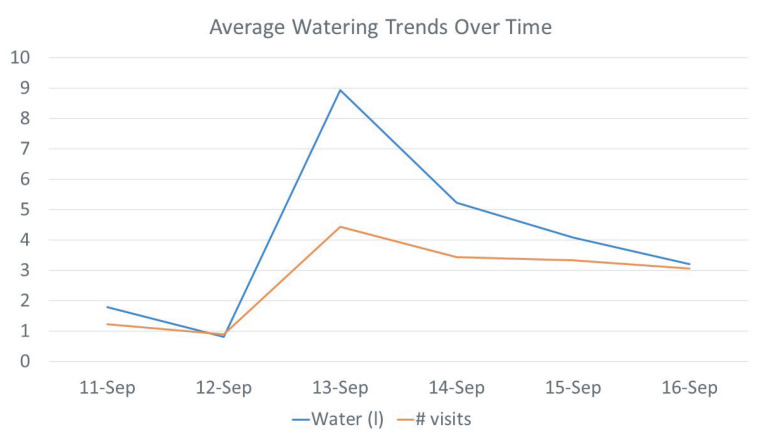
Daily average watering behavior of the drinking station in terms the number of visits and water intake (liters).

The water intake sensor works together with the animal identification sensor to monitor each drinking event of an individual animal. Figure 13 demonstrates individual water intake per animal and Figure 14 plots the number of visits of each animal that was recorded daily. Data from five days were illustrated. It took approximately two days for the calves to become accustomed to drinking out of the system, with 1.24 average visits on the first day of the study versus 3.07 average visits per animal by the fifth day. Each individual cow is labeled a specific animal ID, which was plotted using different colors in the figure. Figure 15 shows the average of the water intake and visits of all animals. As watering behavior is a key component to overall water intake for the individual animal, such data generated from the system can not only measure actual water consumption, but can also be used for important watering behavior which may associate with the animal growth and other health-related issues.

**Figure 16:**
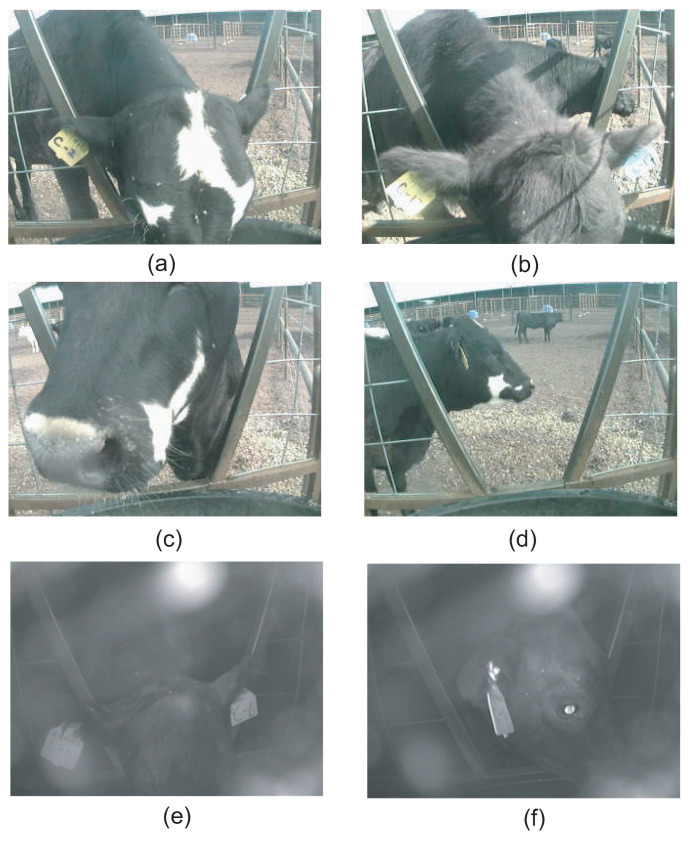
Image taken by the cameras triggered by motion detector: (**a**) Animal ID readable. (**b**) Animal ID not unreadable due to light. (**c**) Animal ID blocked. (**d**) False trigger of the camera with no drinking event. (**e**) Evening drinking event with animal ID readable. (**f**) Evening event with animal ID unreadable.

During a drinking event, the motion detector triggers the camera to take pictures. Figure 16 shows example pictures recorded by the camera and stored in the SD card. The pictures are used along with the RFID system to identify the individual cow. Each cow has an RFID tag attached to its ear. The animal ID is also marked on the ear tag. Although the RFID reader can automatically detect the ID, sometimes it misreads it, so it still needs help from the camera systems to identify the animal manually. Figure 16a shows a successful example that the animal ID can be read from the camera. However, sometimes it is difficult to read due to light conditions, as shown in Figure 16b, or because it is blocked, as shown in Figure 16c. The motion camera may make mistakes triggering the camera when there are actually no drinking events. For example, animals walking close to the motion detector may trigger the camera Figure 16d. The camera also works in the evening. Animal IDs can be read from evening pictures Figure 16e, although sometimes it would be blocked as shown in Figure 16f. The size of each picture is small, which is between 12 and 28 kB, so that the SD card can store the raw images for more than two weeks.

**Figure 17:**
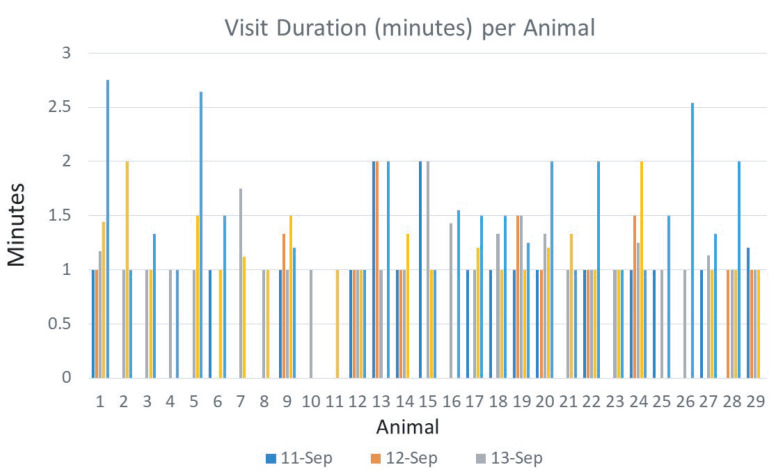
Recorded visit duration by individual animals.

**Figure 18:**
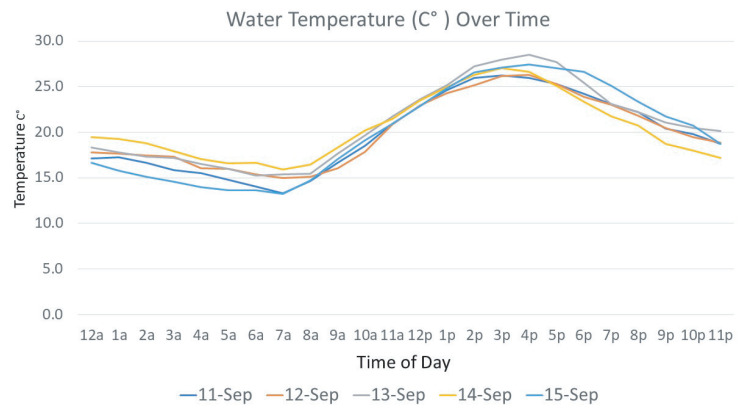
Recorded water temperature data with different day and time.

The watering behavior is analyzed by the visit duration per animal and the specific drinking time during the day. It is also related to the water quality such as temperature, pH value, turbidity, and mineral levels of water. The current system detects the drinking duration in the timing resolution of minutes, which could be improved in later systems to the timing resolution of seconds. Furthermore, the currently deployed system only measures the water temperature, which would be extended into pH value and other chemical components of water in future systems. Figure 17 shows how long each animal stayed at the water source. The time was broken into average minutes per day; however, some animals were noted to stand at the water source as long as 10 min without drinking. Water temperature could impact water consumption as well. Figure 18 shows the average water temperature collected by the system. Tracking daily water temperature will not only reflect air temperature changes but could also demonstrate optimum temperature for maximum water intake and watering behavior. Table 1 presents a few lines of raw data stored in the SD card between 8 am and 9 am on August 6 at Station 4 on the East side of the Tucumcari site.

## 5. Discussion

This work presented a sensor-based data collection and analysis system to track water intake for both domestic cattle and wild animals. The system records the water consumption of individual animals using the water level sensor. It also records the duration of each drinking event. The animal identification is detected using motion detectors, cameras, and RFID systems. Water temperature and turbidity are also measured and recorded. The system also measures the total water consumption and sends an alert signal if more water should be pumped from the external water source. The overall sensing system is controlled by multiple microcontroller units with communications through ethernet cables. All data are stored digitally and automatically using the SD card interface. The system is deployed in reliable weatherproof circuit boxes.

The key advantage of the system is that it provides an important tool to track individual animal water consumption. These data could be used to evaluate each animal’s health and for herd management. If any individual animal demonstrates an abnormal drinking pattern, early detection and intervention could be triggered to save cost. Water temperature and quality recording also help to avoid water-related animal health issues. Moreover, the water intake measurement helps water saving as better knowledge of the decomposition of water consumption could be obtained. Furthermore, the system provides unique access to study wild animal drinking events in remote grazing sites. The automatic sensing and data recording system helps data analysis and overall animal monitoring. Table 2 summarizes the system features and compares them with other recent similar systems. The primary difference between our proposed system and other similar systems is that it can be deployed in remote sites without confinement. The proposed system also provides images of the drinking events, which is uniquely helpful for recording potential wild animals.

The measurement error of the system comes from the individual sensors. For example, the temperature sensor has a resolution of 0.1 degree and the water depth sensor has a resolution of 0.1 cm. Such error is good enough for our application. As usually the drinking events do not last very long, disturbances such as water evaporation or raining are not considered in the current system, but may be included in the future studies. The water intake measurement has error due to the unstable of the water surface. There are many different readings during the drinking event. We are taking the medium value to balance the accuracy and data storage. The next system will combine the water level reading with the scale reading that measures the weight of the water tank to improve the reading accuracy. The current system has a few shortcomings that would be addressed in future improvements. For example, the animal detection system is sometimes not reliable. The motion detector may be triggered by other motions besides cows and wild animals as some pictures triggered by the motion detector are showing no animals at the drinking station. The RFID system is also not 100% reliable. It may miss some animal visits. The water sensors lack the capability to detect the mineral levels of water. Moreover, currently, the system data collection and transmission are done manually by people fetching the SD card, which is difficult as usually people travel to the remote site once every two weeks. Furthermore, the duration and life are unknown as it is an ongoing research. However, we are closely monitoring the condition of operation. Data from the RFID systems, motion sensor-based cameras, and water level monitors are analyzed to make sure that the real drinking events is detected and the animal ID is detected. A valid drinking event happens only when the water level monitor records water consumption. We recorded several pictures from the camera showing no animals at the drinking station and some RFID events without images from the camera. Both cases have no water consumption. Therefore, we believe the cross-validation could demonstrate the accuracy of the system. A more accurate measurement is to deploy a continuous monitoring surveillance system to verify the drinking events manually, which we are planning in the future experiments. Therefore, more future work could be performed to enhance system functionality and performance.

Currently, the system can detect the drinking event including the animal ID, the water consumption, and temperature of the water. The current system does not have the capability to process data from individual sensors to identify sensor failures. For example, the system cannot verify the detected animal ID from the RFID system using the image data. The water turbidity sensor was not enabled and would be added in the later experiments. The system can be further improved by future work from different aspects. For example, the communication system from the remote site could be replaced by satellite communication or dedicated long-range telecommunication. Currently, the data were stored locally in the SD card as the remote site was not covered by the cellular data service. We are trying to deploy data links at the remote site. Once the data link is deployed, we can create a wireless sensor network. At this moment, the remote site has no wireless communication means (cellular or internet). Therefore, the current system does not address the central failure problem. In future work, we plan to add more backup data storage in the system. The animal identification system would benefit from a machine learning-based animal face recognition system in order to alleviate the problem of error detections. Water quality refers to water temperature and water turbidity. More water sensors could be included in the system such as pH sensors specific chemical sensors, for example, sensors to detect Sulfur, Calcium, and Iron, which are the most common problems in New Mexico waters. Weight scales can also be implemented at the drinking station to measure and record the animal’s weight during the drinking event. The improved sensing system may be connected to control systems to perform better water management. For instance, shutting the water supply off if water contamination is detected due to acid rain or other reasons. The system may be combined with weather systems to have an early preparation of weather changes such as heavy rains or high temperatures. Furthermore, the drinking behavior data such as visit time and visit duration of the domestic animal could be applied to train machine learning algorithms to identify the abnormal features of animal health status [47,48,49,50,51]. Early detection and intervention could help save costs related to animal health issues. In summary, more technological advancements are expected to further facilitate water management for animal agriculture. The current system is designed to perform the basic function of water intake monitoring. The presented experimental results are only a demonstration of the system performance. In future work, we also plan to improve the error analysis during system optimization.

## 6. Conclusions

In this work, a water intake monitoring system with animal ID detection and water quality tracking was implemented. The system monitors each drinking event in terms of animal identification, water consumption, drinking behavior, and water quality. The system also can send an alert signal when the water level is too low. The animal identification was performed using aRFID and motion-sensing based camera. The water level is measured by distance sensors. The proposed system was implemented in the remote grazing site. It performs data acquisition and logging automatically. The data are stored on SD cards and displayed in real-time on an LCD screen. Sensing and logging are controlled by low-power microcontrollers. The overall system can be powered by either solar cells or AC power. Besides monitoring livestock drinking behavior, the system could also monitor wild animals’ water intake, which would provide new insights for wildlife conservation. The current system automatically records the drinking behavior of 29 cows in a two-week duration in the remote ranch, which could not be done without the proposed system. The system has the potential to save water consumption while providing access to animal health monitoring, which is important for future animal agriculture applications.

## Figures and Tables

**Table 1:** Data recorded in the SD card.

Date	Time	Temperature (${}^{\circ }$C)	Water Depth Difference (cm)	Water Consumed (L)
6-Aug	8–18a	23.4	0.2	1.45
6-Aug	8–18a	23.4	0.4	3.29
6-Aug	8–22a	23.5	0.2	1.25
6-Aug	8–23a	23.6	0.3	2.00
6-Aug	8–40a	23.9	0.7	5.41
6-Aug	8–44a	24.0	0.1	1.06
6-Aug	8–57a	24.1	0.6	4.92

**Table 2:** Comparison between this work and recently published systems.

	This Work	[43]	[44]	[45]	[46]
Type	Academic	Academic	Academic	Industrial	Industrial
Year	2021	2020	2019	2018	2020
Method	Image + RFID + Depth sensor	RFID + Water flow sensor	RFID + Accelerometer	Flow meter	RFID
Animal ID	Yes	Yes	Yes	No	Yes
Indoor Confinement Only	No	Yes	Yes	Yes	Yes
Potential Wildlife Usage	Yes	No	No	N/A	No

## Data Availability

Not applicable.
